# High FLT3 expression indicates favorable prognosis and correlates with clinicopathological parameters and immune infiltration in breast cancer

**DOI:** 10.3389/fgene.2022.956869

**Published:** 2022-09-08

**Authors:** Rui Chen, Xinyang Wang, Jingyue Fu, Mengdi Liang, Tiansong Xia

**Affiliations:** ^1^ Department of Breast Surgery, First Affiliated Hospital, Nanjing Medical University, Nanjing, China; ^2^ Department of Thyroid and Breast, The Second Affiliated Hospital of Nantong University, Nantong, China

**Keywords:** FLT3, tumor infiltrating lymphocytes, the tumor microenvironment, immunotherapy, breast cancer, prognosis, DNA methylation

## Abstract

**Purpose:** Breast cancer is a highly heterogeneous malignancy, seriously threatening female health worldwide and inducing higher mortalities. Few have the studies evaluated Fms-like TyrosineKinase-3 (FLT3) in prognostic risk, immunotherapy or any other treatment of breast cancer. Our study focused on investigating the function of FLT3 in breast cancer.

**Patients and methods:** Based on transcriptome and methylation data mined from The Cancer Gene Atlas (TCGA), we explored the clinical features of FLT3 expression in 1079 breast cancer samples. RT-qPCR in cell lines and tissue samples was used to verify the expression difference of FLT3. Kaplan–Meier survival analysis and cox regression models were employed for screening of FLT3 with potential prognostic capacity. Subsequently, functional analysis of the co-expressed genes was conducted using Gene Ontology (GO), Kyoto Encyclopedia of Genes and Genomes (KEGG), and gene-set enrichment analysis (GSEA). The correlation between FLT3 expression and tumor immune infiltration was jointly analyzed with estimate, ssGSEA, TIMER, and TISIDB. Then we employed checkpoint-related molecules, immunophenoscore (IPS), and tumor mutation burden (TMB) to assess the efficacy of immuno-checkpoint inhibitors (ICIs). Pearson correlation coefficient was employed to exam the association between DNA methylation and FLT3 expression.

**Results:** FLT3 displays an elevated expression in breast cancer than normal pairs and is significantly associated with multiple clinical characteristics like age, menopause status, histological type, pathological stage, and molecular subtype as well as increased overall survival (OS). Additionally, FLT3 is a favorable independent prognostic factor. GO, KEGG, and GSEA suggested that FLT3 was associated with diversified immune-related features. FLT3 expression is correlated with the abundance of various immune cells namely CD4+T cell, CD8+ T cell, myeloid dendritic cell, and neutrophil as well as immune inhibitors especially CTLA4, which is positively correlated with FLT3 expression. Moreover, TMB displayed a negative correlation with FLT3 expression while IPS showed adverse tendency. Ultimately, the methylation of FLT3 downregulates the gene expression and closely binds to a few clinical parameters.

**Conclusion:** FLT3 can be used for prognostic prediction and is relevant to immune infiltration in breast cancer. FLT3 may pave the way for future novel immunotherapies.

## 1 Introduction

In accordance with GLOBOCAN 2020 estimation, the morbidity of breast cancer (BC) has surpassed lung cancer in female patients worldwide with 2.3 million newly diagnosed cases (11.7%) (Sung et al., 2021). BC can be divided into four subtypes, triple-negative being the least common type (about 15%), luminal A and luminal B being the most prevalent types (about 70%), and HER2-positive taking up the rest of 15–20 percent. Thus, BC is an intricate disease and comprehensive treatment is fairly important. Besides surgery and radiotherapy, systemic adjuvant therapies including chemotherapy, endocrine therapy, targeted therapy and immunotherapy can be used in the face of molecular type and clinicopathology staging. Despite substantial progress in adjuvant treatment recently, difficulties to monitor therapeutic efficacy as well as prognosis remain urgent issues to be solved (Loibl et al., 2021).

The tumor microenvironment (TME) indicates normal tissue components around tumor cells, containing extracellular matrix (ECM), stroma, lymphatic vascular networks, fibroblasts and multiple immune cells (Chen et al., 2015). The immune infiltrating cells in the TME are deeply intertwined with tumor generation, aggression and metastasis (Binnewies et al., 2018). According to recent studies, tumor immune infiltration is closely bound up with prognosis and gradually becoming the focus of immunotherapy (Zhang and Zhang, 2020).

Fms-like TyrosineKinase-3 (FLT3) is a fundamental component of the type III tyrosine kinase receptor family. As a surface protein of FLT3LG, FLT3 is restrictedly expressed on hematopoietic stem cells (HSCs) and figures prominently in cell survival, proliferation and differentiation (Matthews et al., 1991). FLT3 mutations are the most frequently identified gene aberrations found in acute myeloid leukemia (AML) and indicate adverse clinical outcomes (Kiyoi et al., 2020).

No former study has dug into the mechanism of FLT3-associated immune infiltration of BC. Currently, we utilized the cancer genome atlas (TCGA), an open-access databank which harbors clusters of clinical parameters as well as transcriptome data, to explore the issue above. Firstly, the expression profiles of FLT3 were analyzed together with clinical variables to examine the relationship between the two. A similar analysis was carried out at the DNA methylation level of FLT3. Furthermore, our results verified the positive prognostic significance of increased FLT3 expression among BC patients. Gene Ontology (GO), Kyoto Encyclopedia of Genes and Genomes (KEGG) pathway analysis and gene set enrichment analysis (GSEA) were used to probe into the possible molecular function of FLT3. We explored the correlation between FLT3 and tumor-infiltrating immune cells (TILs) along with immunoregulatory factors via TIMER, TISIDB database and ssGSEA. These findings would aid us in better grasping the part that FLT3 played in breast carcinogenesis and the underlying mechanisms associated with tumor-immune interactions. Meanwhile, FLT3 is expected to predict the survival outcomes and therapeutic effects of BC patients.

## 2 Materials and methods

### 2.1 Data acquisition and processing

UCSC Xena (https://xenabrowser. net/) (Cline et al., 2013) provides analyzed data in TCGA, from where we downloaded mRNA-sequencing, DNA methylation, and clinical phenotype results of BC patients. The following criteria were used for exclusion: 1) the histological diagnosis was not standard, 2) the specimens did not have complete clinical data available. The datasets were pre-processed by using R (version 4.1.2) and Perl (version 5.30.2) software.

### 2.2 Gene expression and DNA methylation analysis

The ensemble gene identifiers in the FPKM data were switched to classic gene symbols using Perl script and the data on duplicate gene expression were averaged. Bioinformatic parameters like age, gender, menopausal condition, histological type, pathological staging and molecular subpopulation acquired from 1,079 primary BC samples and 99 paired adjacent non-tumorous tissue samples were used for further research. We classified BC into PAM50-based intrinsic subtypes: luminal A, luminal B, HER2-enriched, basal-like, and normal-like subtype (Wang et al., 2021). Further, the expression data of FLT3 in different phenotypes were analyzed and visualized using ggplot2 (Gustavsson et al., 2022) and ggpubr (Xia and Li, 2020) R packages. Meanwhile, DNA methylation analysis of FLT3 was conducted along with data on clinical features and vital status.

### 2.3 Survival analysis

Profiles of overall survival (OS), disease-specific survival (DSS), disease-free interval (DFI), and progression-free interval (PFI) were obtained from TCGA survival data. The Prognostic outcome was visualized in the form of Kaplan–Meier (K-M) curves using the “survival” package (Zeng et al., 2022) in R software (version 4.1.2), in which Cox’s proportional hazards model was generated as well to figure out whether FLT3 is an independent prognostic factor of BC. To be specific, univariate Cox regression analysis was used for the primary screening of the survival-related variables and only significant ones (*p* < 0.05) would be included in the subsequent multivariable analysis. The hazard ratio30 with 95% confidence intervals (CI) and log-rank *p*-value was also calculated. We also explored the prognostic value of FLT3 in BC on the prognoscan website (http://dna00.bio.kyutech.ac.jp/PrognoScan/index.html) (Zheng et al., 2020).

### 2.4 DEGs and gene enrichment analysis

The data of 1,079 samples were separated into low and high expression groups in accordance with the median value of FLT3 expression. Based on differentially expressed genes (DEGs_ (| log2FC | ≥ 0.5, FDR <0.05), the “clusterprofiler” package was utilized to perform GO and KEGG analysis (Huang et al., 2022). *p* values were adjusted with the BH method. GSEA is an intricate algorithm to see the chosen gene sets showing statistical distinctions between different biological conditions (Subramanian et al., 2005). In our study, an organized list of all genes based on their associations with the FLT3 expression was obtained by using GSEA, which later expounds evident survival differences between high-and low-FLT3 groups. Gene set permutations were repeated 1,000 times at every single analysis. Gene sets with a discovery rate (FDR) < 0.05 were recognized to be considerably enriched.

### 2.5 Characterization of the tumor microenvironment

On the basis of the BC gene set, the “estimate” R package was utilized for TME score calculation (Wang et al., 2020). The assessment of TME was divided into four clusters (stromal score, immune score, estimate score and tumor purity). The potential relevance of FLT3 with immune/stromal scores was analyzed and displayed by violin plot. The single sample gene set enrichment analysis (ssGSEA) (Jin et al., 2021) performed by the “gsva” (Hänzelmann et al., 2013) package was applied to grade the enrichment of 28 immune cells in BC. The TIMER (https://cistrome.shinyapps.io/timer/) database was used to evaluate the abundance of tumor-infiltrating immune cells (TILs) (Li et al., 2017). Tumor-infiltrating immune cells containing CD8^+^ T cell, CD4^+^ T cell, B cell, macrophage, neutrophil, and dendritic cell are closely geared to TME. Thus, we employed the TIMER2.0 to dig out the correlation of FLT3 expression with TILs. TISIDB (http://cis.hku.hk/TISIDB) (Ru et al., 2019), a user-friendly web interface containing a great deal of tumor-associated immune cells, immunomodulators, chemokines and immunotherapies, verified the connection between FLT3 and TME.

### 2.6 Tumor mutation burden

Tumor mutation burden (TMB) is defined as the number of somatic mutations per megabase of interrogated genomic sequence (Negrao et al., 2021) and the mutation data of 986 BC patients were obtained from the TCGA database. The total number of mutations divided by the size of the target coding region is equal to the tumor mutation load.

### 2.7 Immunophenoscore analysis

Immunophenoscore (IPS) was used to predict the efficacy of ICIs and measured according to the four major types of genes determining immunogenicity (Charoentong et al., 2017). The IPS is ranging from 0 to 10 and a higher IPS represents a better efficacy and stronger immunogenicity. The IPSs of TCGA BC suffers were acquired from The Cancer Immunome Atlas (TCIA) (Wang et al., 2022).

### 2.8 Cell culture

Human mammary epithelial cell line (MCF-10A) and human BC cell lines (ZR-75-1, MCF-7, BT-474, SKBR-3, MDA-MB-231, and BT-549) were obtained from American Type Culture Collection (ATCC) (Manassas, VA, United States ) and cultured in DMEM (Gibco, United States ) with 10% fetal bovine serum, penicillin (100 U/mL) and streptomycin (100 mg/ml) at 37°C with 5% CO_2_.

### 2.9 Clinical samples

Fourteen patients who underwent surgery in the First Affiliated Hospital with Nanjing Medical University between July 2021 and December 2021 were enrolled in the study and provided signed informed consent. Pairs of BC and adjacent normal tissue were obtained immediately after resection and preserved in liquid nitrogen. The research was approved by the Medical Ethics Committee of Nanjing Medical University, the First Affiliated Hospital.

### 2.10 RNA extraction and quantitative real-time polymerase chain reaction

Trizol reagent (Takara, Japan) was utilized for total RNA extraction from cell lines and tissue samples. The HiScript Q RT SuperMix (Vazyme, China) was used for complementary DNA (cDNA) synthesizing. Ultimately, qRT-PCR was performed using AceQ qPCR SYBR Green Master Mix (Vazyme, China). β-actin was used as the internal control for the relative expression of mRNA, which was calculated by the 2^−ΔΔCT^ method. The specific primer sequences used were as follows: β-actin-F: ATT​GCC​GAC​AGG​ATG​CAG​AA; β-actin-R: GCT​GAT​CCA​CAT​CTG​CTG​GAA; FLT3-F: AGG​GAC​AGT​GTA​CGA​AGC​TG; FLT3-R: GCT​GTG​CTT​AAA​GAC​CCA​GAG.

### 2.11 Statistical analysis

Statistical datasets acquired from TCGA were all conducted by R-4.1.2. The correlations between the clinicopathological parameters, FLT3 expression and DNA methylation degree of FLT3 were analyzed using logistic regression. Moreover, the K-M curve and COX regression analysis were conducted to evaluate the prognostic value of FLT3. When *p* < 0.05, a statistically significant distinction was considered.

## 3 Results

### 3.1 Differential expression of fms-like tyrosinekinase-3

**FIGURE 1:**
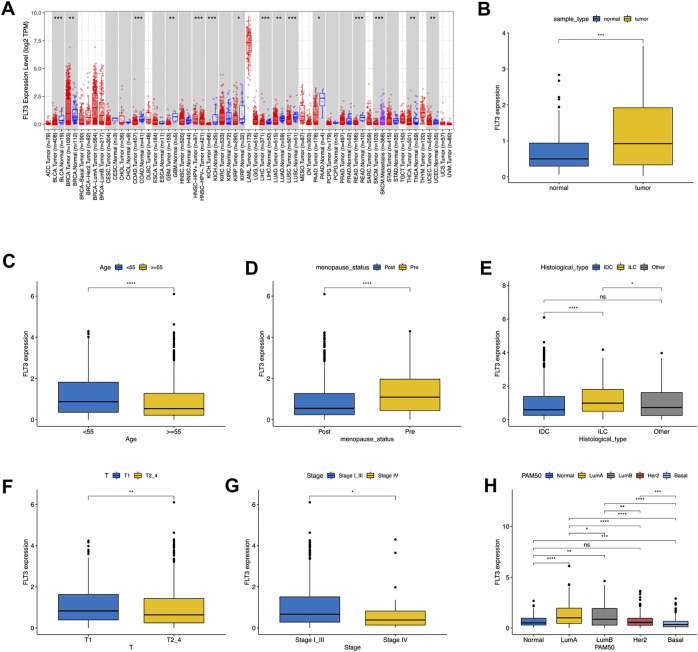
Associations between FLT3 expression and clinical–pathological variables in BC. The level of FLT3 expression in different tumor types from TCGA data analyzed in TIMER. FLT3 was highly expressed in breast cancer (BRCA), and lowly expressed in bladder cancer (BLCA), cervical cancer (CECS), bile duct cancer (CHOL), colon adenocarcinoma (COAD), head and neck cancer (HNSC) tissue, kidney chromophobe (KICH), kidney clear cell carcinoma (KIRC), Liver hepatocellular carcinoma (LIHC), lung adenoma (LUAD), lung squamous cell carcinoma (LUSC), pancreatic adenocarcinoma melanoma (PAAD), pheochromocytoma and paraganglioma (PCPG), prostate adenocarcinoma (PRAD), rectum adenocarcinoma (READ), stomach adenocarcinoma (STAD), thyroid cancer (THCA) and endometrioid cancer (UCEC) and **(A)**. High expression of FLT3 was observed in tumor tissue both in all samples **(B)**. FLT3 expression was analyzed in female and male **(C)**, postmenopausal and premenopausal **(D)** patients, with different histological types **(E)**, including invasive ductal carcinoma (IDC), invasive lobular carcinoma (ILC), and all other specific types, different tumor size **(F)** (T1 versus T2, T3, and T4), different pathological TNM stage**(G)** (Stage I, II and III versus Stage IV) and different PAM50 molecular subtypes **(H)**, T1, tumor ≤20 mm in greatest dimension; T2, Tumor >20 mm but ≤50 mm in greatest dimension; T3, Tumor >50 mm in greatest dimension; T4, Tumor of any size with direct extension to the chest wall or the skin (ulceration or macroscopic nodules); invasion of the dermis alone does not qualify as T4; N0, no regional lymph node metastasis; N1, metastasis in 1–3 axillary lymph nodes; N2, metastasis in 4–9 axillary lymph nodes; and N3, metastasis in 10 or more axillary lymph nodes; M0, without distant metastasis; and M1, with distant metastasis; Stage I, T1N0M0; Stage II, T0-1N1M0, T2N0-1M0, T3N0M0; Stage III, any T stage with N2M0 or N3M0, T4N0M0, T3-4N1M0; Stage IV, any T or N stage with M1. The asterisks represent the statistical *p*-value (ns: *p* > 0.05, *p ≤ 0.05, **p ≤ 0.01, ***p ≤ 0.001, ****p ≤ 0.0001).

First, we examined the expression level of FLT3 in different tumors and paired normal samples using the TIMER database. It revealed that FLT3 was highly expressed in BC compared to normal tissue, while other tumors were just the opposite (Figure 1A). On account of the data from TCGA, we concluded that FLT3 displayed higher expression in BC tissue than in the respective control tissue (Figure 1B). As shown in Supplementary Table S1, with 1079 BC patients taken into account, we found that FLT3 transcription level correlated notably with age, menopausal status, histological type, T stage, pathological stage and molecular subtype. Two age sets (the elder set, and the younger set) have been identified in 1079 BC patients according to the cut-off value of 55 years old, the elder set displayed a lower FLT3 expression level than another (Figure 1C). As revealed in Figure 1D, FLT3 expression decreased after menopause. When it comes to histological type, BC can generally be divided into three, namely invasive ductal carcinoma (IDC), invasive lobular carcinoma (ILC) and other type. Our results implied that ILC had the highest FLT3 expression level and the difference between IDC and ILC was rather significant (Figure 1E). Additionally, increased FLT3 expression was noted in the early T stage and vice versa (Figure 1F). Similar results were obtained in pathological TNM stage while there is no statistical meaning between FLT3 expression and N or M stage (Figure 1G). For the convenience of statistics, we simplified the PAM50 molecular subtype to luminal-like, HER2-enriched, basal-like and normal-like. Boxplot in Figure 1G displayed that the basal-like group had the lowest FLT3 expression level and the luminal-like group had the highest (Figure 1H).

### 3.2 Fms-like tyrosinekinase-3 was an independent favorable prognostic factor

**FIGURE 2:**
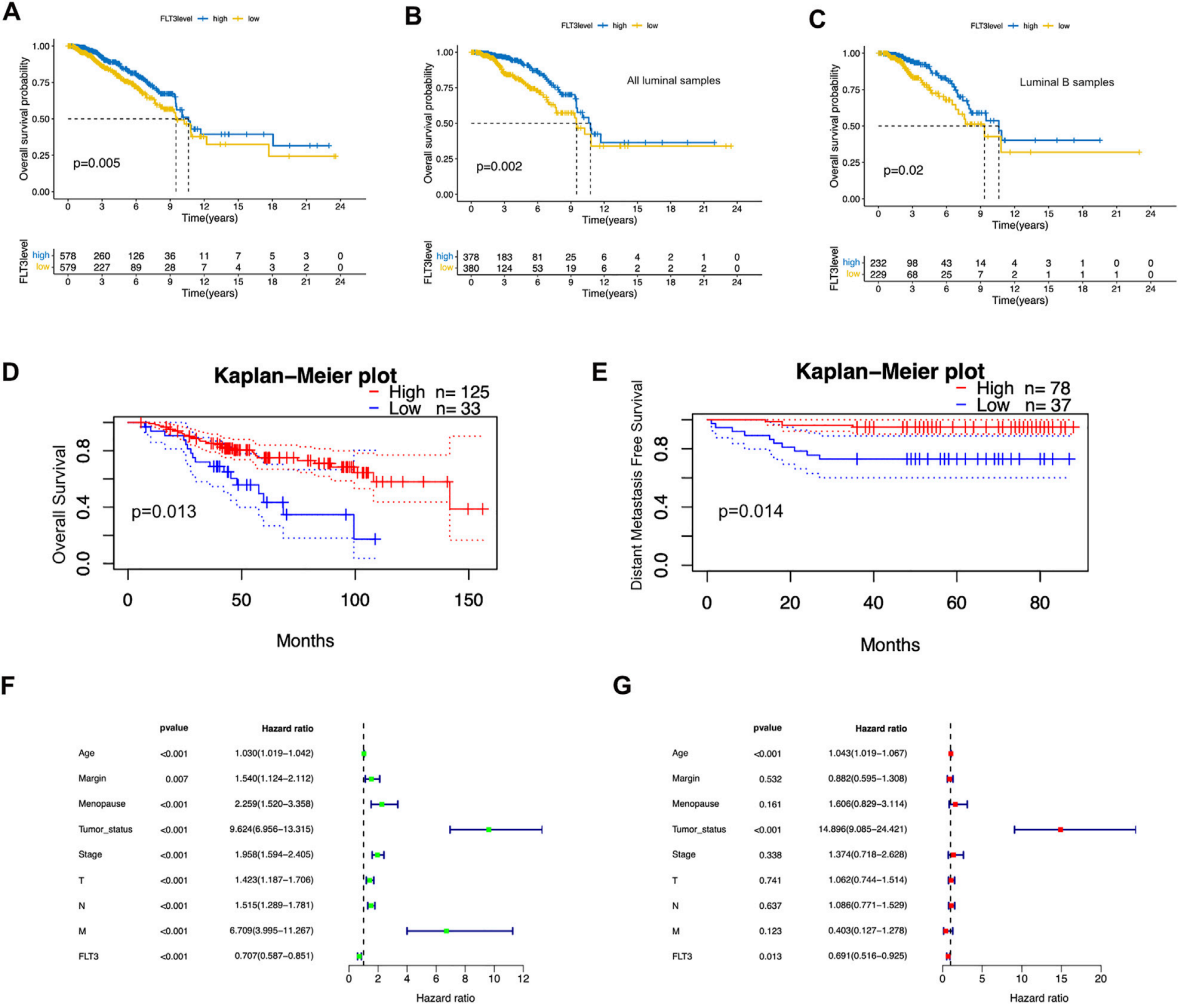
Prognostic value of FLT3 expression in BC. **(A)** High FLT3 expression was associated with a favorable OS in BC patients using Kaplan–Meier plotter. **(B,C)** High FLT3 expression was inferred as a good OS in all luminal samples and all luminal B samples. **(D,E)** The upregulation of FLT3 infers longer OS and DMFS on the website of prognoscan. **(F,G)** Forest plots show the association between FLT3 expression and clinicopathological features using univariate and multivariate COX hazard analysis.

To better comprehend the prognostic potential of FLT3 expression in BC, we conducted Kaplan-Meier survival analysis in terms of overall survival (OS), progression-free interval (PFI), disease-free interval (DFI) and disease-specific survival (DSS). The results showed that high FLT3 expression degree was conspicuously associated with increased overall survival (OS) while the others showed no statistical significance (Figure 2A). As indicated in Figures 2B,C, overexpression of FLT3 has good prognostic value in all luminal samples and luminal B subtype. Figures 2D,E show the result from the website of prognoscan that a high level of FLT3 expression in BC indicates longer overall survival (OS) and distant metastasis free survival (DFMS), which is consistent with our results. We next employed the COX proportional hazard regression model to verify the role of FLT3 expression in evaluating patients’ vital status. At univariate analysis, FLT3 expression level turned out to be an independent prognostic biomarker. Several clinical parameters such as age (HR = 0.706, 95% CI:0.586-0.851, *p* < 0.001), tumor status (HR = 9.623, 95% CI:6.956-13.314, *p* < 0.001), menopause condition (HR = 2.259, 95% CI:1.519-3.357, *p* < 0.001) and margin status (HR = 1.54, 95% CI:1.123-2.111, *p* = 0.007) as well as pathological stage (T stage: HR = 1.423, 95% CI:1.187-1.706, *p* < 0.001; N stage: HR = 1.515, 95% CI:1.289-1.781, *p* < 0.001; M stage: HR = 6.709, 95% CI:3.995-11.266, *p* < 0.001; TNM stage: HR = 1.957, 95% CI:1.593-2.404, *p* < 0.001) correlate closely with prognosis of BC patients (Figure 2F). Further multivariate survival analysis was shown in Figure 2G, FLT3 expression (HR = 0.69, 95% CI:0.515-0.925, *p* = 0.013) independently impacts prognosis while age (HR = 1.042, 95% CI:1.018-1.066, *p* < 0.01) and tumor status (HR = 14.895, 95% CI:9.085-24.421, *p* < 0.01) were significant characteristics that affect the survival of BC patients. In general, high FLT3 expression level is an independent prognostic factor of favorable significance.

### 3.3 Enrichment analysis

**FIGURE 3:**
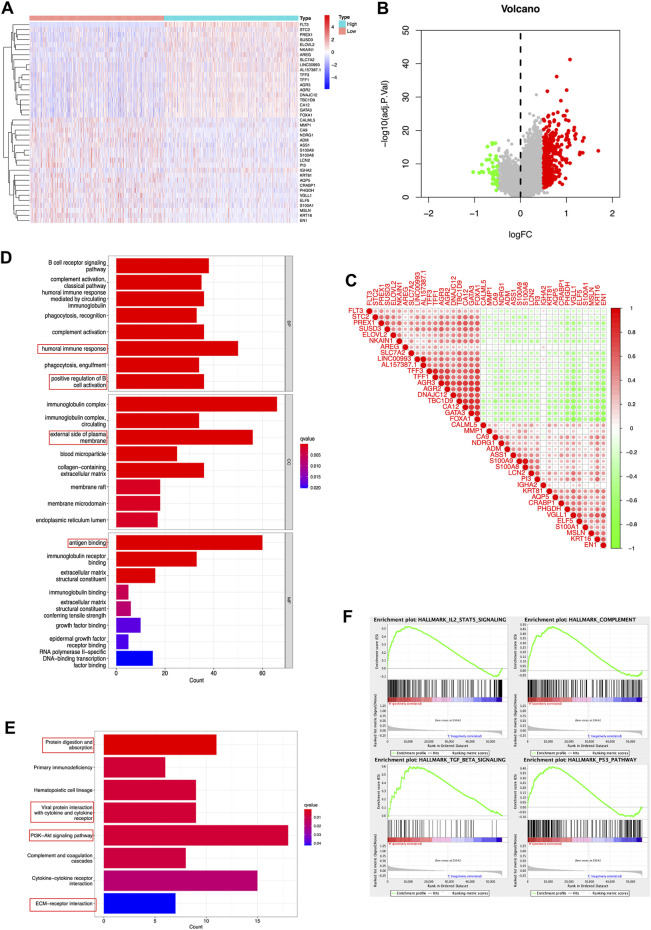
DEGs and enrichment analyses of FLT3 in BC. **(A)** Heatmap and **(B)** volcano plot show the DEGs in high and low FLT3 expression patients. **(C)** Correlation of FLT3 and the top 40 co-expressed genes. **(D,E)** GO enrichment and KEGG pathway analysis of FLT3 in BC. The red box highlights the pathways correlated with BC and immune infiltrates. **(F)** The GSEA results showed that the terms “complement”, “IL2-STAT5 signaling”, “TGF-BETA signaling” and “p53 pathway” were differentially enriched in BC samples with high FLT3.

In a gesture to identify the potential molecular function of FLT3 in breast carcinogenesis, we performed differential expression gene analysis (DEGs) and visualized the results as the heatmap and volcano plot (Figures 3A,B). FLT3 co-expressed genes were depicted in Figure 3C. DEGs between the high-risk and low-risk groups were used to perform GO enrichment and KEGG pathway analysis. In Figure 3D, five biological processes, cellular components or molecular functions: immunoglobulin complex, antigen binding, external side of plasma membrane, humoral immune response and positive regulation of cell activation were remarkably enriched in the GO dataset. KEGG pathway analysis uncovered that FLT3 was conspicuously associated with protein digestion and absorption, hematopoietic cell lineage, viral protein interaction with cytokine and cytokine receptor, PI3K-Akt signaling pathway and cytokine-cytokine receptor interaction (Figure 3E), the results of which hint that FLT3 is greatly related to immune pathways. For the sake of predicting the molecular function of FLT3 in breast carcinogenesis, GSEA was performed on datasets with high and low expression of FLT3. 38 out of 50 gene sets were remarkably upregulated in the high FLT3 phenotype. The most markedly gathered at NOM *p* < 0.05, FDR <0.25 signaling pathways were “complement”, “IL2-STAT5 signaling”, “TGF-BETA signaling” and “p53 pathway”, whose results were depicted in Figure 3F.

### 3.4 The relationship between fms-like tyrosinekinase-3 expression and immune infiltration

**FIGURE 4:**
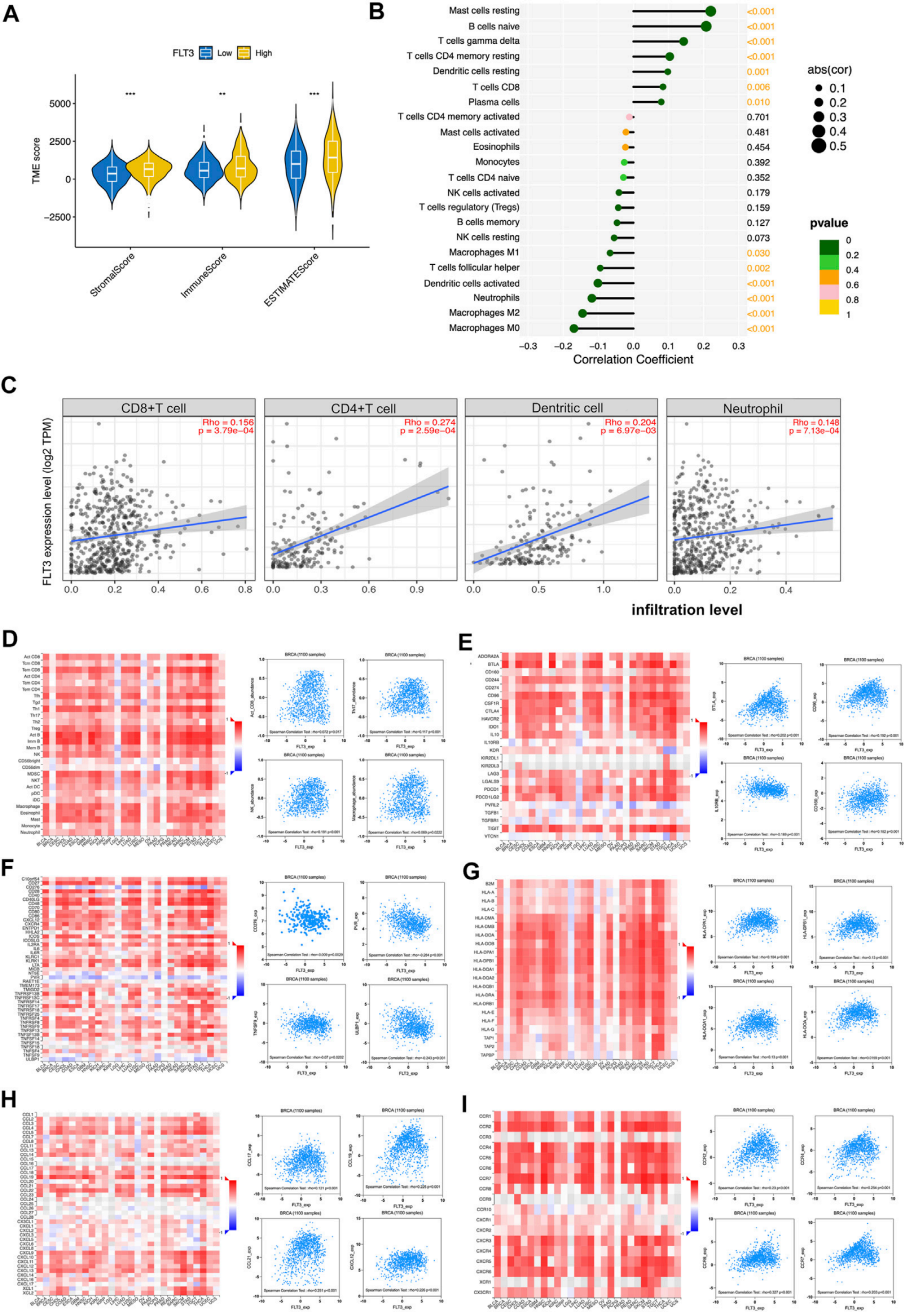
Associations of the FLT3 expression level with tumor immune infiltration in BC. **(A)** The correlation between FLT3 expression and TME scores, which was subdivided into stromal scores, immune scores, and ESTIMATE scores. **(B)** The correlation between the abundance of 28 immune cells and FLT3 expression. **(C)** The correlation of FLT3 expression with infiltration levels of CD4+T cell, CD8^+^ cell, dendritic cell, and neutrophil in BC is available on the TIMER 2.0 database. **(D)** Correlations between the abundance of tumor-infiltrating lymphocytes (TILs) and FLT3 (plus the four anti-tumor immune cells which have positive correlation with FLT3) in the TISIDB database. **(E–G)** Correlations between immunomodulators and FLT3 (plus the four immunomodulators with the highest correlation, respectively) in the TISIDB database. **(H,I)** Correlations between chemokines (or receptors) and FLT3 (plus the four chemokines (or receptors) which highly correlates with anti-tumor immune cells, respectively) in the TISIDB database. The asterisks represent the statistical *p*-value (ns: *p* > 0.05, *p ≤ 0.05, **p ≤ 0.01, ***p ≤ 0.001, ****p ≤ 0.0001).

Immune infiltration in tumor can conduce progression and recurrence so as to affect clinical outcomes of cancer patients. We applied the ESTIMATE algorithm to evaluate stromal and immune cells in BC. The figure showed that patients with high-FLT3 expression get higher immune scores compared with patients with low-FLT3 expression. A similar trend was observed in stromal and estimate scores (Figure 4A). When characterizing the abundances of disparate immune cell types with ssGSEA, we found that the infiltration degree of NK cell, CD8+T cell, CD4+T cell, and macrophage increased significantly in BC samples with high FLT3 expression (Figure 4B). To conclude, BC samples with higher FLT3 expression tend to be infiltrated with more antitumor immune cells. To testify to this, TIMER and TISIDB database to explore immune cell sets infiltrated in the TME. From TIMER, the abundance of CD4+T cell (Rho = 0.201, *p* = 5.07e-03), CD8+T cell (Rho = −0.142, *p* = 7.17e−06), myeloid dendritic cell (Rho = 0.26, *p* = 2.76e−02), and neutrophil (Rho = 0.148, *p* = 7.13e−04) increased as the FLT3 expression upregulates (Figure 4C). From TISIDB, we discovered that FLT3 was strongly correlated to activated CD8^+^ T cell (Rho = 0.072, *p* = 0.017), NK (Rho = 0.191, *p* = 1.97e-10), macrophage (Rho = 0.069, *p* = 0.0222), and neutrophil (Rho = 0.168, p = 2e-08) (Figure 4D). To further explore the FLT3-related immune processes in BC, the database was employed to analyze the relationship between FLT3 and different immuno-modulators, which can be separated into immune-inhibitors, immune-stimulators and MHC molecules. Figure 4E reveals the relationship between FLT3 expression and immune-inhibitors, including BTLA, IL10RB, CD160, and CD96. Figure 4F reveals the relationship between FLT3 expression and immune-stimulators, including ULBP1, TNFRSF13B, PVR, and ENTPD1. Figure 4G reveals the relationship between FLT3 expression and MHC molecules, including HLA-DOA, HLA-DQA1, HLA-DPB1, and HLA-DPA1. Figure 4H illustrated the association between FLT3 and chemokines like CCL17, CCL19, CCL21, and CXCL21. Figure 4I illustrated the association between FLT3 and chemokines receptors like CCR2, CCR4, CCR6, and CCR7. Obviously, the expression of these chemokines for NK, DC, and T lymphocytes increased as the expression level of FLT3 elevated. With all the findings above, we can speculate that high-FLT3 expression indicating a favorable prognosis can probably attribute to immune infiltration.

### 3.5 Fms-like tyrosinekinase-3 associated with immune checkpoint remedy

**FIGURE 5:**
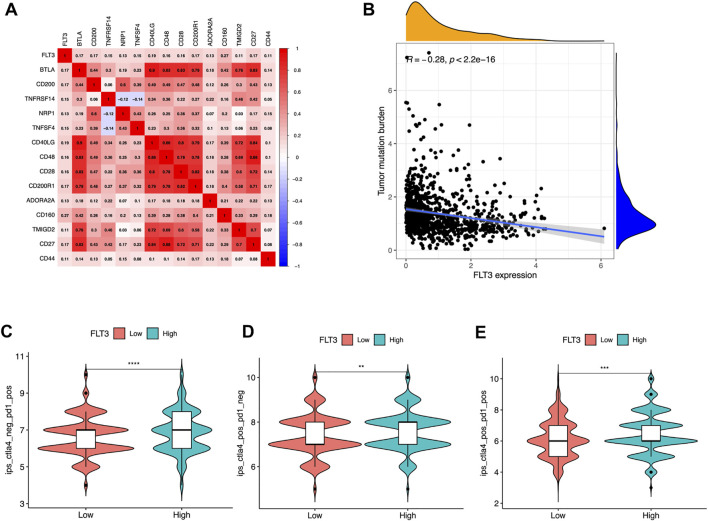
Associations of FLT3 with immune-related genes. **(A)** Correlations between FLT3 and immune checkpoint molecules. Red is positive, and blue is negative. The numbers in the circle represented the correlation value and all the *p*-value <0.05. **(B)** Correlation between FLT3 expression and tumor mutation burden. Violin plots showing the showing the FLT3 expression between **(C)** IPS-PD1/PD-L1/PD-L2, **(D)** IPS-CTLA4, and **(E)** IPS- CTLA4 and PD1/PD-L1/PD-L2 scores and FLT3 expression in patients of The Cancer Genome Atlas breast cancer. The asterisks represent the statistical *p*-value (ns: *p* > 0.05, *p ≤ 0.05, **p ≤ 0.01, ***p ≤ 0.001, ****p ≤ 0.0001).

Recent scholars have reported that the microenvironment of tumor has guiding significance for evaluating the efficacy of immune checkpoint therapy (Hegde et al., 2016). Further, to broaden the cognition of the correlation between FLT3 and immunotherapy, we investigated the connections between FLT3 expression and immune checkpoint-related genes, the result of which was depicted in Figure 5A. Several immune checkpoint molecules including BTLA, CD200, TNFRSF14, NRP1, TNFSF4, CD40LG, CD48, CD28, CD200R1, ADPRA2A, CD160, TMIGD2, CD27, and CD44 were positively related to FLT3 mRNA expression (correlation value = 0.17, 0.17, 0.15, 0.13, 0.15, 0.19, 0.16, 0.17, 0.13, 0.27, 0.11, 0.17, and 0.11; all *p*-values are <0.001). Figure 5B illustrated that tumor mutation burden (TMB) has a negative correlation with FLT3 expression. It was verified that IPS has the potential to predict the treatment response to ICIs (Charoentong et al., 2017). The IPS data was acquired from the TCIA webpage to explore the relevance of FLT3 expression to IPS. The FLT3-highly-expressed subgroup had a statistically higher IPS- CTLA4 or PD1/PD-L1/PD-L2 score or IPS- CTLA4 and PD1/PD-L1/PD-L2 score (Figures 5C–E).

### 3.6 Methylation negatively connected with mRNA expression

**FIGURE 6:**
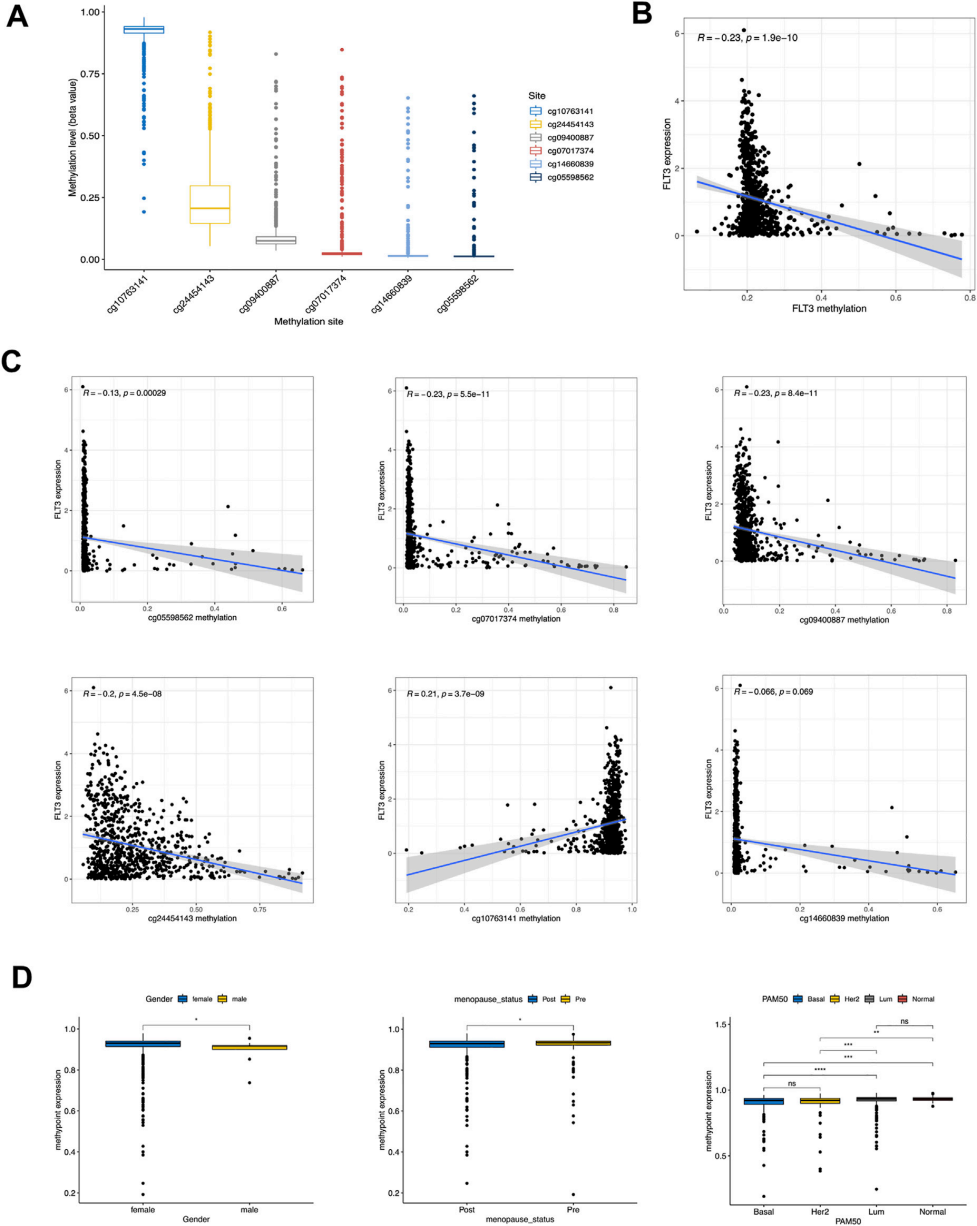
Correlation of DNA methylation level with FLT3 expression and clinical features. **(A)** Methylation level of 8 methylation site in FLT3. **(B)** Correlation of FLT3 expression with gross methylation level. **(C)** Correlation of FLT3 expression with different methylation sites (cg05598562, cg07017374, cg09400887, cg24454143, cg10763141 and cg14660839). **(D)** Methylation level of cg24454143 site was analyzed with gender, different menopausal status and PAM50 molecular subtypes. The asterisks represent the statistical *p*-value (ns: *p* > 0.05, *p ≤ 0.05, **p ≤ 0.01, ***p ≤ 0.001, ****p ≤ 0.0001).

In a gesture to better understand the expression difference of FLT3, we conducted further analysis at methylation levels. Figure 6A showed the methylation levels of dissimilar sites of FLT3 DNA, with the site cg10763141 possessing a high methylation degree and the others possessing a low. Figure 6B demonstrated that FLT3 methylation level decreased along with the increase of FLT3 expression level. We explored the relationship between FLT3 expression and diverse methylation sites respectively. Subsequently, negative correlations with the methylation degree of cg05598562, cg07017374, cg09400887, cg24454143 along with the opposite trend in cg10763141 were depicted in Figure 6C. The distinction between FLT3 expression and cg14660839 is not obvious. Moreover, clinical relevance analysis was conducted with respect to the methylation degree of each site. Figure 6D turned out that the methylation degree of cg24454143 is significantly correlated with gender, menopause status and PAM50 subtype, which displayed an adverse tendency compared to the previous FLT3 expression levels in similar clinical parameters.

### 3.7 Fms-like tyrosinekinase-3 mRNA expression difference in cell lines and tissue samples

**FIGURE 7:**
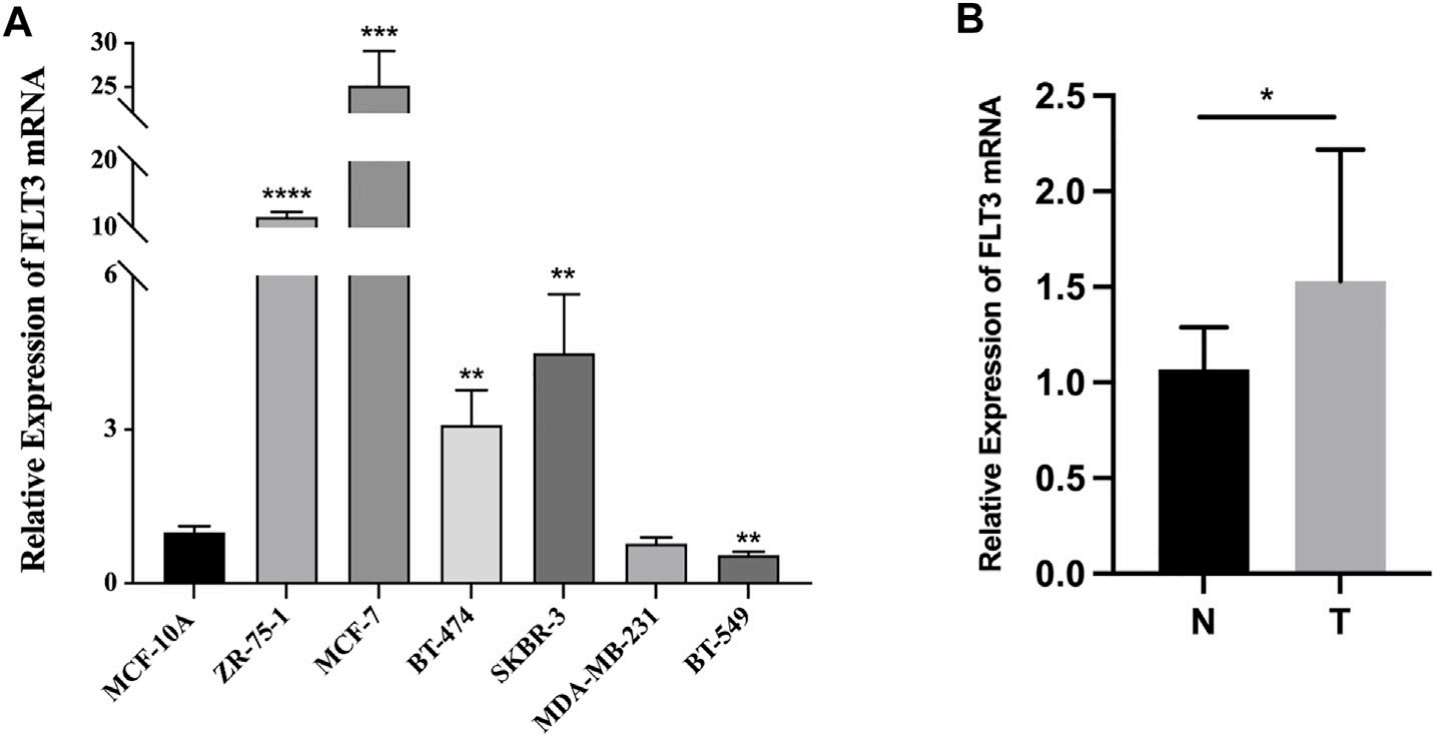
The mRNA expression of FLT3 in BC cell lines and tissue samples. **(A)** Relative expression level of FLT3 in MCF-10A, ZR-75-1, MCF-7, BT-474, SKBR-3, MDA-MB-231, and BT-549 cell lines. **(B)** The qRT-PCR result which compared the expression levels of FLT3 in tumor and para-carcinoma tissue of BC patients. The asterisks represent the statistical *p*-value (ns: *p* > 0.05, *p ≤ 0.05, **p ≤ 0.01, ***p ≤ 0.001, ****p ≤ 0.0001).

Quantitative RT-PCR in BC cell lines and tissue samples was conducted to testify to the expression difference of FLT3 in the TCGA database. BC cell lines (ZR-75-1, MCF-7, BT-474, and SKBR-3), non-triple-negative BC subtypes, expressed significantly higher levels of FLT3 than the common human mammary epithelial cell line. Meanwhile, the triple-negative subtype cell line (BT-549), displayed lower FLT3 expression than the normal human mammary epithelial cell line (Figure 7A), which was consistent with our results in Figure 1H. Compared with paired paracancerous tissue, FLT3 was highly expressed in BC tissue (Figure 7B).

## 4 Discussion

Known as a surface protein, Fms-like tyrosine kinase 3 (FLT3) is a component of the receptor tyrosine kinase family. FLT3 is normally expressed on hematopoietic stem cells (HSCs) and activated upon integrating with FLT3 ligand, which further activates a series of downstream signaling cascades, resulting in cellular hyperplasia and containment of apoptosis and differentiation (Carow et al., 1996). Generally, FLT3 is well studied in acute myeloid leukemia (AML). FLT3 mutations are the most prevalent genetic aberrations discovered in acute myeloid leukemia (AML), accounting for 30% and represent a high potential of relapse and poor prognosis (Mrózek et al., 2007; Kennedy and Smith, 2020). Hence, an array of FLT3 targeted molecules have been evaluated and several progress in FLT3-defined therapies has been made (Zhao et al., 2022). One research found FLT3 expression upregulated in axillary lymph node (ALN) metastases when compared to primary lesions in triple-negative BC (TNBC) (Srour et al., 2020). A few studies verified the potent antitumor activity of FLT3 ligand (FLT3L) in the murine BC model, which can open up new ideas for the development of tumor vaccines (Chen et al., 1997; Braun et al., 1999). Neoadjuvant In Situ Immunomodulation (ISIM) regimen comprised of intra-tumoral administration of Flt3L, local radiotherapy, and *in situ* TLR3/CD40 stimulation, followed by surgical resection in murine metaplastic BC model shows great therapeutic potential for advanced BC (Oba et al., 2021).

Analysis of transcriptome data from the TCGA portal revealed higher FLT3 expression in BC than in normal pairs and our experimental outcomes have validated this. We accessed clinical information to find that elevated expression of FLT3 correlated with early tumor stage and increased survival. Later, the survival analysis demonstrated that high FLT3 expression was an independent survival determinant of BC (BC). The enrichment analysis indicated that FLT3 participate in immune-associated pathways like complement pathway, which displayed anti-tumor capacity in mouse BC models (Roumenina et al., 2019). It is reported that the upregulation of STAT5 can increase the overall survival of ER-positive BC patients (Halim et al., 2020). Moreover, the number and activity of tumor-infiltrating lymphocytes (TILs) can impact the survival of patients in several cancers (Gu et al., 2020). Our results illustrated that increased expression of FLT3 can propel distinct clusters of anti-tumor cells infiltrating in BC. Immune cell subpopulations related to adaptive immunity including activated CD8^+^ T cell, Tem and Tcm CD8^+^ cell, and Tem CD4^+^ cell infiltration were rather noteworthy, which were verified to indicate improved survival (Paijens et al., 2021). Simultaneously, chemokines and receptors are also critical contributors to TME by drastically increasing the infiltration of immune cells (Chen et al., 2020; Liu et al., 2020). There is a positive association between FLT3 expression and chemoattractant for NK, DC, and T cell, which counts a lot in combating malignancies and thus improving prognosis (Zhang and Zhang, 2020). Taken together, the results above robustly manifested that FLT3 expression level could be a prognostic biomarker for BC. Whereas impediments to a cure arise not just from cancer itself, TME can also exert vital influences on multiple stages of tumor proliferation and progression. TILs constitute the most important part of the TME (Chen et al., 2014). With insights into TILs recently, the paradigm of immunotherapies has switched from targeting tumor cells to immune cells (Pilipow et al., 2021). Inhibitors of PD-1/PD-L1 and CTLA4 targeting lymphocytes (Emens et al., 2021) were existing immunotherapies for BC, which focus on the triple-negative subtype. In practical, few patients can benefit from ICIs, thus we need to find effective ways to evaluate the efficacy. Our results turned out that dissimilar immune checkpoint molecules were positively associated with FLT3 expression. High TMB suggests that more neoantigens are produced, and T cells liberated by immune checkpoint inhibitors are more probably to identify neoantigens, thereby achieving the effect of attacking and killing tumors. In this way, TMB can predict the efficacy of ICIs (Negrao et al., 2021). A previous study characterized BC as low TMB and without full response to immunotherapies (Kandoth et al., 2013), which is consistent with our conclusion. Whereas, Sara et al. held the opinion that the significance of TMB in BC remains unclear for lacking sufficient investigation (Ravaioli et al., 2020). In this study, the high-FLT3-expression group had statistically higher IPS-CTLA4 and PD1/PD-L1/PD-L2 scores, suggesting that patients with high-FLT3 expression would develop a better response to combination remedy. In all, FLT3 expression level may have the potential to predict the response of immunotherapies.

DNA methylation attaches importance to a series of biological processes. Accumulating evidence has revealed that DNA methylation can bring transformation in chromosome structure, the molecular conformation of DNA, DNA stability and the way DNA interacts with proteins, thereby regulating gene expression (Wang et al., 2021). A negative correlation between DNA methylation and FLT3 expression was revealed in our study. At the site cg24454143, methylation degree is statistically obvious in terms of clinical variables like gender, menopause status and PAM50 subtype, which is contrary to the former analysis in RNA expression level.

Although our analysis has elucidated that high FLT3 expression is closely connected to longer survival and potent antitumor immunity, which seems sensible but requires further experimental validation. To be specific, more endeavors such as immunohistochemical staining on our clinical samples and additional analysis of the multiple clinical parameters and survival profiles are needed.

## 5 Conclusion

Taken together, it is believed that FLT3 could strengthen our understanding of prognosis in BC patients and help promote the development of novel immune-strategies and achieve optimal clinical efficacy. Overall, the potential influence and mechanism of FLT3 in BC deserves further exploring.

## Data Availability

The datasets presented in this study can be found in online repositories. The names of the repository/repositories and accession numbers can be found in the article/Supplementary Material.
