# Supplementary material for: High FLT3 expression indicates favorable prognosis and correlates with clinicopathological parameters and immune infiltration in breast cancer
Source: Front Genet. 2022 Sep 8;13:956869. doi: 10.3389/fgene.2022.956869 (PMC9499177; doi:10.3389/fgene.2022.956869)
Supplement: Supplementary file 1 [file Table1.DOCX]

Supplementary Material

# Supplementary Table 1

Relationship between FLT3 expression level and clinical characteristics in breast cancer.

| Clinical Characteristics | Total | FLT3 | | P value |
| --- | --- | --- | --- | --- |
|  |  | **High** | **Low** |  |
| Age |  |  |  |  |
| <55 | 489 | 289(49.15%) | 200(33.96%) | <0.05 |
| ≥55 | 688 | 299(50.85%) | 389(66.04%) |  |
| Gender |  |  |  |  |
| Female | 1164 | 581(98.81%) | 583(98.98%) | 0.9975 |
| Male | 13 | 7(1.19%) | 6(1.02%) |  |
| Histological type |  |  |  |  |
| IDC | 856 | 394(67.01%) | 462(78.44%) | <0.05 |
| ILC | 204 | 133(22.62%) | 71(12.05%) |  |
| Other | 117 | 61(10.37%) | 56(9.51%) |  |
| Menopause status |  |  |  |  |
| Post | 740 | 329(66.33%) | 167(33.67%) | <0.05 |
| Pre | 254 | 167(33.67%) | 87(17.47%) |  |
| Margin status |  |  |  |  |
| Close | 23 | 14(2.55%) | 9(1.64%) | 0.5484 |
| Negative | 1034 | 515(93.98%) | 519(94.54%) |  |
| Positive | 40 | 19(3.47%) | 21(3.83%) |  |
| Tumor status |  |  |  |  |
| With tumor | 138 | 67(11.69%) | 71(12.66%) | 0.6854 |
| Tumor free | 996 | 506(88.31%) | 490(87.34%) |  |
| PAM50 |  |  |  |  |
| Basal | 201 | 51(8.67%) | 150(25.47%) | <0.05 |
| Her2 | 113 | 48(8.16%) | 65(11.04%) |  |
| LumA | 301 | 190(32.31%) | 111(18.85%) |  |
| LumB | 470 | 265(45.07%) | 205(34.8%) |  |
| Normal | 92 | 34(5.78%) | 58(9.85%) |  |
| T |  |  |  |  |
| T1 | 300 | 168(28.67%) | 132(22.45%) | <0.05 |
| T2 | 686 | 334(57%) | 352(59.86%) |  |
| T3 | 144 | 71(12.12%) | 73(12.41%) |  |
| T4 | 44 | 13(2.22%) | 31(5.27%) |  |
| N |  |  |  |  |
| N0 | 545 | 279(48.1%) | 266(46.26%) | 0.1304 |
| N1 | 401 | 211(36.38%) | 190(33.04%) |  |
| N2 | 127 | 53(9.14%) | 74(12.87%) |  |
| N3 | 82 | 37(6.38%) | 45(7.83%) |  |
| M |  |  |  |  |
| M0 | 994 | 500(98.43%) | 494(97.05%) | 0.2074 |
| M1 | 23 | 8(1.57%) | 15(2.95%) |  |
| Stage |  |  |  |  |
| 1 | 193 | 114(19.39%) | 79(13.41%) | <0.05 |
| 2 | 672 | 336(57.14%) | 336(57.05%) |  |
| 3 | 268 | 121(20.58%) | 147(24.96%) |  |
| 4 | 21 | 7(1.19%) | 14(2.38%) |  |
