# Supplementary material for: High FLT3 expression indicates favorable prognosis and correlates with clinicopathological parameters and immune infiltration in breast cancer
Source: Front Genet. 2022 Sep 8;13:956869. doi: 10.3389/fgene.2022.956869 (PMC9499177; doi:10.3389/fgene.2022.956869)
Supplement: Supplementary file 2 [file Table2.DOCX]

PCR数据

<https://www.jianguoyun.com/p/DdDrUwMQrsXUChjn68UEIAA>

R代码

https://www.jianguoyun.com/p/DWyKUecQrsXUChjo68UEIAA

原始数据

<https://www.jianguoyun.com/p/DQErgnMQrsXUChjv68UEIAA>

<https://www.jianguoyun.com/p/DbJ9whQQrsXUChiU7MUEIAA>

<https://www.jianguoyun.com/p/DcnQSm0QrsXUChi77MUEIAA>

https://www.jianguoyun.com/p/DakuFHMQrsXUChjB7MUEIAA
